# The Impact of an Electronic Patient Bedside Observation and Handover System on Clinical Practice: Mixed-Methods Evaluation

**DOI:** 10.2196/11678

**Published:** 2019-03-06

**Authors:** Alexandra Lang, Mark Simmonds, James Pinchin, Sarah Sharples, Lorrayne Dunn, Susan Clarke, Owen Bennett, Sally Wood, Caron Swinscoe

**Affiliations:** 1 Trent Simulation and Clinical Skills Centre Nottingham University Hospitals NHS Trust Nottingham United Kingdom; 2 Nottingham University Hospitals NHS Trust Nottingham United Kingdom; 3 Faculty of Engineering University of Nottingham Nottingham United Kingdom; 4 NHS Digital Leeds United Kingdom

**Keywords:** health information technology, early warning score, mobile health, staff workload, clinical deterioration, patient safety, mixed methods

## Abstract

**Background:**

Patient safety literature has long reported the need for early recognition of deteriorating patients. Early warning scores (EWSs) are commonly implemented as “track and trigger,” or rapid response systems for monitoring and early recognition of acute patient deterioration. This study presents a human factors evaluation of a hospital-wide transformation in practice, engendered by the deployment of an innovative electronic observations (eObs) and handover system. This technology enables real-time information processing at the patient’s bedside, improves visibility of patient data, and streamlines communication within clinical teams.

**Objective:**

The aim of this study was to identify improvement and deterioration in workplace efficiency and quality of care resulting from the large-scale imposition of new technology.

**Methods:**

A total of 85 hours of direct structured observations of clinical staff were carried out before and after deployment. We conducted 40 interviews with a range of clinicians. A longitudinal analysis of critical care audit and electronically recorded patient safety incident reports was conducted. The study was undertaken in a large secondary-care facility in the United Kingdom.

**Results:**

Roll-out of eObs was associated with approximately 10% reduction in total unplanned admissions to critical care units from eObs-equipped wards. Over time, staff appropriated the technology as a tool for communication, workload management, and improving awareness of team capacity. A negative factor was perceived as lack of engagement with the system by senior clinicians. Doctors spent less time in the office (68.7% to 25.6%). More time was spent at the nurses’ station (6.6% to 41.7%). Patient contact time was more than doubled (2.9% to 7.3%).

**Conclusions:**

Since deployment, clinicians have more time for patient care because of reduced time spent inputting and accessing data. The formation of a specialist clinical team to lead the roll-out was universally lauded as the reason for success. Staff valued the technology as a tool for managing workload and identified improved situational awareness as a key benefit. For future technology deployments, the staff requested more training preroll-out, in addition to engagement and support from senior clinicians.

## Introduction

### Background

Patient safety literature has long reported the need for early recognition of deteriorating patients, with health strategy stating it as a health care priority [1-4]. Early warning scores (EWSs) are commonly implemented as “track and trigger,” or rapid response systems for monitoring and early recognition of acute patient deterioration.

We have presented an evaluation of a hospital-wide transformation from paper-based patient observations to mobile handheld device–enabled electronic observations (eObs) and electronic handover (eHandover) data collection in a large UK teaching hospital. Over 5500 mobile devices were deployed to over 6000 staff across 80 wards. The innovative technology allows real-time, automatic information processing at the patient’s bedside, with the aim of improving the efficacy of EWSs in practice and provide greater visibility of key patient data.

This study evaluated the deployment using objective measures and subjective evaluation of changes in clinical practice in addition to the overview of benefits realization based on analysis of hospital data sets.

Independent human factors researchers evaluated the technology implementation through pre- and postdeployment observations and staff interviews. Data extracted from the eObs and eHandover software and other supporting information technology (IT) systems provide evidence of the impact of this intervention on admissions to critical care and other benefits to the organization.

The combination of these datasets provides valuable insight into how a health information technology (HIT) intervention has affected care provision and patient safety in a large UK teaching hospital.

### Background to Paper-Based Early Warning Scores and the Move to Electronic Observations

EWS systems deliver a standardized approach to observation frequency and response based around an aggregated scoring system which characterizes a patients’ physiological acuity. This process involves physiological observations being carried out at the patient’s bedside and a score being calculated (afferent limb). If this score meets the defined criteria, representing a significant abnormality, the observations are communicated and acted upon by appropriate clinical team members (efferent limb) [5]. For the system to be effective, both the afferent *and* efferent limbs need to be efficient, reliable, and timely. The system enables a proactive approach to assessment and recognition of the deteriorating patient, leading to reductions in critical care admissions, mortality, and serious adverse events [6,7].

Historically, EWS processes have been implemented via paper-based charts; however, there is widespread acknowledgement of a variety of contextual reasons which lead to poor adherence to this practice [8,9]. To tackle poor adherence and associated organizational issues, Nottingham University Hospitals (NUH) National Health Service (NHS) Trust commissioned and facilitated the development of an electronic system (Nervecentre Software LtdTM), moving to mobile device access for all clinical staff.

**Figure 1:**
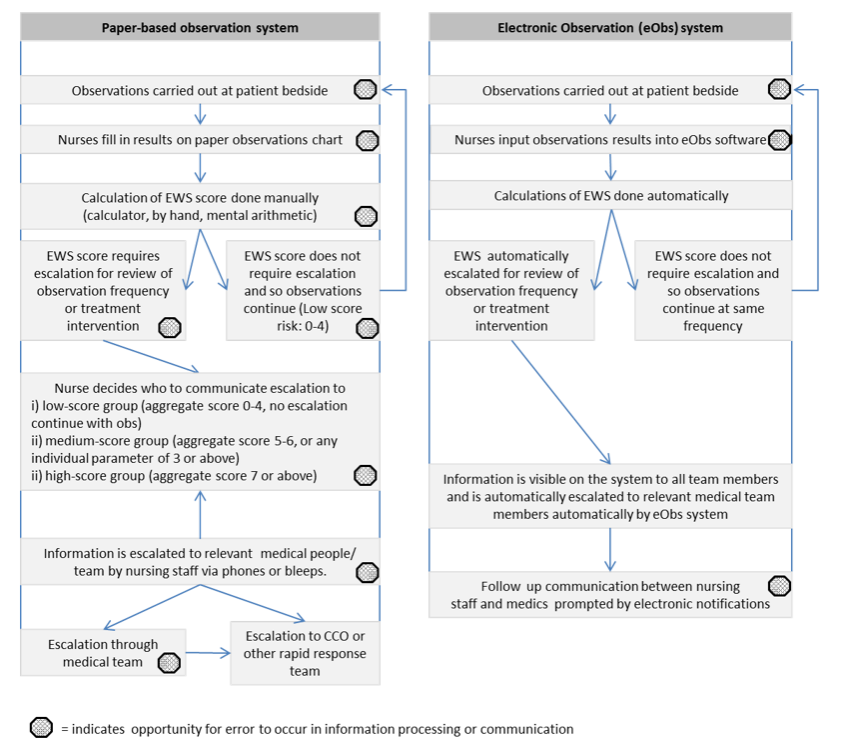
Visual comparison of clinical observation process using paper-based charts (left-hand flow diagram) and the electronic observations system (right-hand flow diagram). eObs: electronic observations; EWS: early warning score; CCO: critical care outreach.

Figure 1 shows the relative complexity of the paper-based system, demonstrating the risk of increased workload and reliance on active communication required of staff in a minimum of 8 decision points during EWS procedures. In comparison, the eObs system has fewer task stages and fewer interpersonal interactions, with automatic system actions replacing the manual score calculation tasks and decision points. To improve adherence, the eObs system also provides a reminder function for the next observation set. The higher number of tasks within the paper-based process increases the risk of potential errors at each stage of processing and communication of information as evidenced by Prytherchy et al [10]. This finding is well established in the literature on errors [11] and specifically the types of errors that occur in paper-based EWSs [12]. There is an increased risk of communication delay, as staff have to prioritize escalation of patient deterioration over other competing tasks. In contrast, the functions of the new electronic system streamline the process and reduce the number of opportunities for degradation of information.

The implementation of the eObs technology in 2015 was distinct from the EWS patient management policy which has been established on paper across NUH since 2008. By aligning precisely to the existing policy, a raised EWS is automatically and immediately escalated to senior clinical staff or the critical care outreach team (CCOT) through mobile instant messaging. The data recorded in the eObs module include all the physiological parameters previously calculated in the paper-based observation charts [13]. The system also allows for “special circumstances models” to be implemented where patient needs differ from standard EWS algorithms (eg, End of Life or known chronically abnormal physiology).

Equally, handover documents have historically been handwritten, nonstandardized, and at risk of being out-of-date, or incorrect, putting patient information and safety at risk [14]. The eHandover solution created a mobile platform to record key patient data in a standardized format, allowing different staff groups to access information where and when they need it. This functionality in eObs and eHandover provides opportunity for consistency, simplicity, and a reduction in the potential for perceptual error.

### Study Context

The study was carried out at a large teaching hospital trust in the United Kingdom, which provides secondary care services for approximately 2.5 million residents and facilitates approximately 1900 beds. Over a period of 9 months, personal mobile devices (iPhones and iPads) and training were provided to over 6000 nurses, doctors, health care assistants, and allied health professionals. The deployment was undertaken by a specialist Clinical Information and Communication Technology (CICT) team. This involved senior and practicing clinical nurses being developed into specialist Information and Communications Technology (ICT) advocates to lead the technology roll-out. Their dual role enabled them to support staff clinically while the general workforce were introduced to and becoming familiar with the eObs system.

This team coordinated the roll-out across 70 adult and children’s wards at 2 hospital sites.

## Methods

### Study Design

The study collected pre- and postdeployment data concerning ward-based work via direct observation of staff before and after the deployment. Interviews and focus groups collected qualitative staff insights into the impact of mobile handheld devices and eObs on nursing and medical practice. Additional data sets were collected from existing hospital systems to give insight into the wider implications of eObs.

Ethical approval was obtained from an appropriate local ethics committee as a service evaluation project.

### Structured Observations

Recruitment of clinical staff was done via flyers and facilitated introductions by the CICT team. Direct structured observations of clinicians were carried out pre- and postdeployment to record staff activities and location within the ward. A total of 23 predeployment and 64 postdeployment (n=87) observation sessions were obtained over 85 hours. Observation sessions lasted between 15 and 120 min. Observers “shadowed” staff, using a bespoke tablet computer app to record activities and locations from exhaustive and exclusive lists. Researchers were not in attendance at the patient’s bedside but observed from a distance, and participants were informed that the observation could be halted at any time.

**Figure 2:**
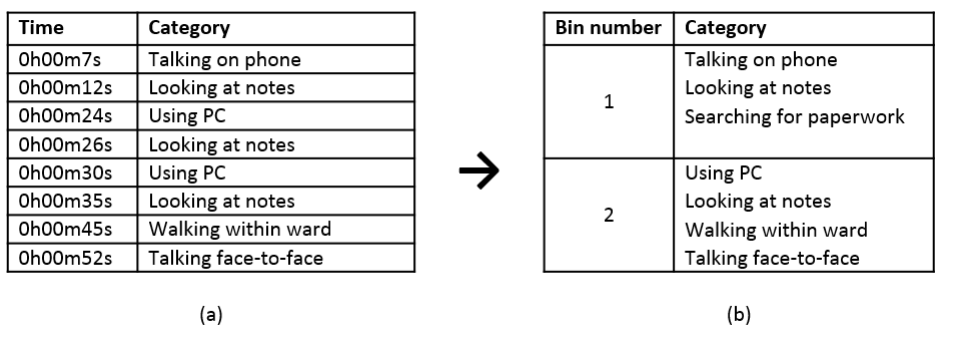
Encoding activity using the structured observation methodology. In (a), 8 sequential activities occur during the 1-min observation window. In (b), a set of unique activities is recorded for each of the two 30-second bins in the observation session. Note that “Looking at Notes” is only recorded once in the first bin despite 2 instances occurring in the first 30 seconds of observation. PC: personal computer.

Observation sessions were divided into 30-second time bins. If an activity was observed in a 30-second bin, it was recorded as 1 observation even if multiple instances of the activity occurred (Figure 2). This method makes the observation of multitasking or rapid task switching possible and provides a measure of the relative distribution of different activities during the observation period.

Predeployment data were collected on a short stay acute medical admissions ward (n=11) and a health care of older person ward (n=13). Postdeployment data were collected on acute medical admissions wards (n=37), medical wards (n=12), and surgical wards (n=15).

Registered nurses were observed for a total of 17 hours predeployment (n=16) and 23.3 hours postdeployment (n=18). Doctors were observed for 10 hours predeployment (n=7) and 35.1 hours postdeployment (n=47). The participating doctors ranged in experience from consultant to Foundation Grade 1 (F1) doctor, which is the general postgraduate bridge between medical school and training for full registration as a medical professional in the United Kingdom.

### Interviews

Recruitment of staff for interviews was carried out via email and poster communications. Semistructured interviews and focus groups explored the impact of technology deployment on personal working practices and also encouraged reflection on the impact on teams, environment, and organization.

A total of 40 interview participants were recruited across a range of nursing and medical roles amounting to 18.5 hours of interview data. The number of interview participants for each staff type is given in Table 1. All interviews were carried out post system deployment, with the staff experience of eObs ranging from 1 week to 5 months.

### Impact Evaluation

A longitudinal analysis of unplanned critical care admissions was derived from the NUH critical care audit dataset. Bed day costs were derived from local single organ high dependency unit (HDU) and 3 organ intensive care unit (ICU) support tariffs.

Review of EWS-related incidents on eObs wards was performed by 2 reviewers independently, from electronically recorded patient safety incident reports (Datix Ltd) from April 2014 to December 2015.

**Table 1:** Stratification of staff interviews.

Clinical role		Number of interviews	Total
**Medical staff**			
	Consultants	5	18
	Registrars	5	
	Locums	1	
	Junior doctors	4	
	CCOT^a^	3	
**Nursing staff**			
	Senior nursing staff	4	12
	Staff nurses	6	
	Health care assistants	2	
CICT^b^ team		7	10
Critical skills educator		1	
Ward managers		1	
Hospital play specialists		1	
Grand total			40

^a^CCOT: critical care outreach team.

^b^CICT: clinical information and communications technology team.

## Results

### Structured Observations

#### Nurses

As expected, an increase in observations of “using smartphone” was detected after the deployment of the eObs technology (2.2% to 6.4% of 30-second bins). However, this change was small when compared with the reduction in time spent interacting with notes and talking on the phone. Table 2 summarizes the changes for key observation categories [15].

A change was also seen in the observed location of nurses undertaking these activities. In particular, a move from the “office” (40.8% to 16.2% of the observed period) to the “nurse’s station” (13.3% to 35.1%) was observed.

A decrease in the number of activities observed in each 30-second bin was observed. This decrease is closely related to a decrease in rapid task switching. The mean number of activities in each 30-second bin decreased from 1.99 (SD 0.04) to 1.66 (SD 0.03).

#### Doctors

The observed changes in the way doctors spend their time were similar to nurses. Smartphone use increased (3.7% to 8.3%) while remaining low relative to the frequency with which interacting with paper notes or desktop PCs was observed. Doctors were also observed spending less time in the office (68.7% to 25.6%) with more time at the nurses’ station (6.6% to 41.7%). Patient contact time more than doubled (2.9% to 7.3%).

#### One-Hour Case Example

Table 3 presents an illustration of how the use of eObs and handheld mobile devices has changed working tasks and locations for clinicians. This example uses a 1-hour exemplar and assumes that each observed activity spanned the entire 30-second observation bin.

**Table:** 

Observation category	Predeployment (% of 30-s observation bins)	Postdeployment (% of 30-s observation bins)
Using Personal Computer (PC) or Computer On Wheels (COW)	23.3	5.1
Looking at notes	36.2	22.3
Writing on notes	26.3	16.0
Talking on phone	8.4	4.0
Using smartphone	2.2	6.4

**Table 3:** One-hour example of changes in doctors and nurses’ clinical tasks and locations of working in the ward owing to electronic observations.

Clinical role			Time
**Doctors**			
	**Task**		
		Average smartphone use	Increase from 2 min to 5 min
	**Location**		
		Time spent in office	Reduce from over 40 min down to less than 16 min
		Time spent at the nursing station	Increase to 21 min
		Time spent with patient	Increase from less than 2 min to over 4 min
**Nurses**			
	**Task**		
		Average smartphone use	Increase from just over 1 min to nearly 4 min
		Use of personal computer (PC) or Computer On Wheels (COW)	Reduce from 14 min down to 3 min
		Looking at notes	Reduce from over 21 min down to less than 14 min
		Writing in notes	Reduce from nearly 16 min down to under 10 min
		Talking on phone	Reduce more than half from 5 min to just over 2 min
		Searching tasks	Reduce by 3.5 min
	**Location**		
		Time spent in office	Reduce from over 24 min to less than 10 min
		Time spent at the nursing station	Increase from 8 to 21 min

### Interviews

A total of 40 staff provided feedback about their experiences of the deployment process, eObs, and mobile devices.

The formation of a specialist clinical team who were trained as ambassadors to lead the deployment was universally lauded as a reason for the successful roll-out. This CICT team was praised for their capacity to multitask, assisting people with the technology while administering clinical care.

During the deployment and early use of the technology, staff reported increased stress and workload, with participants identifying a need for more training in advance of the deployment; however, this was fairly short-lived:

Not all of us had physically got our phone in time so it were all faffing, trying to get the phones charged up and all that kind of technical stuff...we’d not really had time to play with them...I think every one of us felt nervous the morning of it coming and I don’t think we needed to.

Senior nurse

The accessibility of information on the mobile devices appears to have streamlined staff discussions to quickly address treatment pathways by facilitating remote decision making and distributed working for both nursing and medical staff:

The best thing about it is it’s a good record to consult, and certainly patients who’ve had multiple admissions, you can easily go back and see that information from previous admissions, and, unlike paper, it doesn’t get lost.

Junior doctor

Frustration was expressed by junior nursing and medical staff at a perceived lack of engagement with the new system by senior medical personnel (specifically consultants). It was considered that this issue was one of the main barriers to realizing the potential benefits in a ward setting. Several rationales were offered by medical staff (including consultants) to explain the lack of engagement by senior medical staff in the eObs deployment, including the perceived loss of expertise because of the “step change” in practice, the potential for embarrassment associated with use of the new system, or a general reluctance to embrace change.

Junior personnel (medics and nurses) provided an important source of informal device use support to individuals who were struggling to adopt the new system. This support was provided early on and during the weeks and months following the departure of the CICT team from the wards:

If you break it [eObs or phone], even now, it’s a standard joke, we get one of the young staff to fix it.

Senior nurse

“Word of mouth” or “heard it through the grapevine” communications often perpetuated information about eObs and device use throughout the workforce. The staff believed that if this “good practice” could be formally captured and disseminated, it could speed up the rate at which staff experienced benefits from the new system.

Nurse interview data revealed a largely positive response, reporting added value in the form of reassurance of patient health state owing to the real-time eObs information and also awareness of ward capacity. However, there was also an initial perception that the new technology could result in a loss of control for nursing staff and promote a “Big Brother” culture, as automatic escalations meant taking away nursing autonomy. This perspective subsided over time as the real-time automation began to accelerate communications:

Mobile technology has made a huge difference to our working lives. It helps us to manage our workload and feel more in control of what is happening on the ward. It has reinforced the importance of communication between clinicians and has really demonstrated how patient care can be improved.

Ward sister

Over time, nursing staff began to identify how the system could potentially alleviate stress through greater visibility of information. Nurses began to describe the mobile devices as their own “personal tool” for workload management and improved awareness of team capacity:

It is just about making the device work for you...as I’ve got more confident with the device, I have said to my staff, just don’t let it rule your shift and you get it to work for yourself.

Deputy sister

Nursing staff also described the value of handheld devices as a communication tool for use with patients and relatives, whereby the request for information could often be responded to more quickly.

For medics, initial access settings within the eObs system were at odds with current practice in terms of the perception about “consultant-led practice” versus the reality of registrars working independently. During the early deployment of eObs, relationships between consultants and registrars were put under pressure because of the permissions programmed within the system. Clinicians understood the need for policy to underpin the system; however, there was disruption to working practices as these issues were experienced:

...who delivers the cardiac arrest process and decides, well it’s the registrar...so if you are allowing them to make those decisions then to say they can’t alter the parameters is patronising. And it’s again where the trust says ‘all our decisions are consultant made’ but the reality is that’s not true.

Consultant

Medical staff explained how they used the device to “checkup” on patients that they had treated, for clinical reassurance, when they were off duty and had physically left the ward. It was acknowledged that this use of the device should not promote unhealthy practices with regard to work-life balance:

I think it is mainly when I see somebody sick in the ward when on acute medicine...and I just sneak a peek to make sure they are getting better instead of worse...

Registrar

Medical staff believed that the new system had reduced time spent searching within a ward and had facilitated time management, a finding which is consistent with the observation data captured. This related not only to their working practices but also in their reflection of liaison with nursing staff and general hospital organization.

### Longitudinal Impact Data

**Figure 3:**
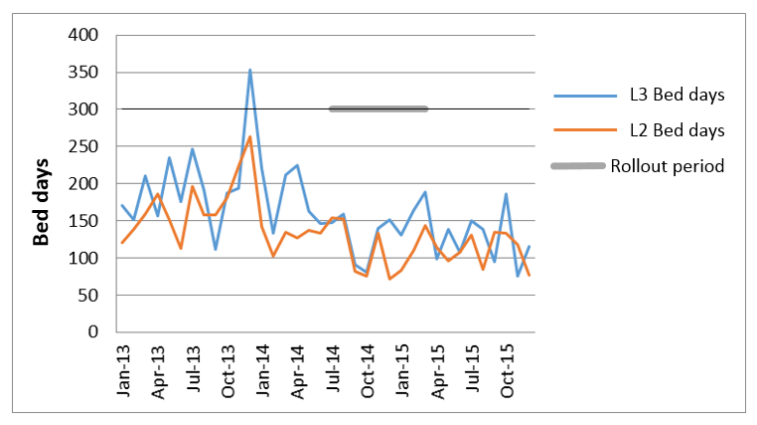
Unplanned admissions to level 2 (high dependency unit) and level 3 (intensive care unit).

The roll-out of eObs has been associated with an approximate 10% reduction in total unplanned admissions to critical care units at NUH from eObs-equipped wards. No substantial change in hospital or critical care bedstock has occurred over this period. This benefit is more marked when critical care level and length of stay are taken into account (Figure 3). Alongside ongoing efforts to improved detection and response to the deteriorating patient [16], the impact of “real-time” communication of EWS and accessibility of patient information via handheld devices appears to be associated with a lower rate of critical care admissions, because of patients receiving more timely care and not requiring referral to those specialist services.

**Figure 4:**
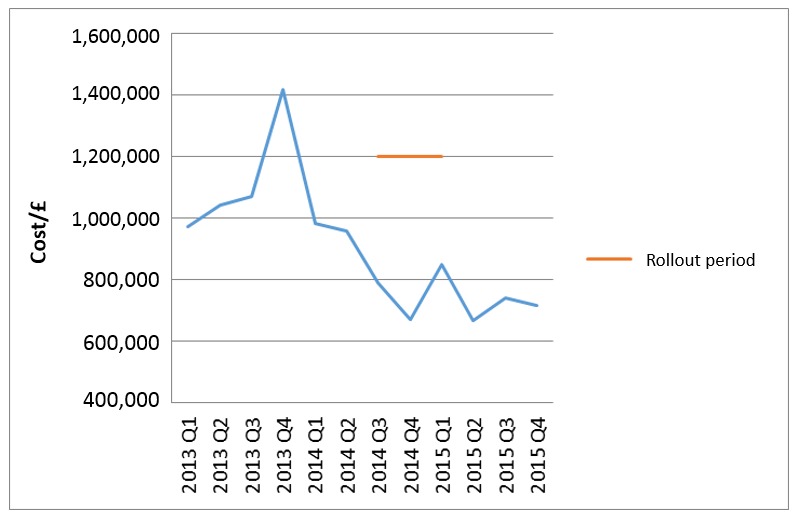
Cost of unplanned critical care admissions.

On the basis of nominal reference costs of £800 per Level 2 (HDU) bed day and £1200 per Level 3 (ICU) bed day, this 10% reduction in critical care admissions equates to an approximate £250K saving per quarter (Figure 4) since deployment.

More detailed cost breakdowns are required to understand the full economic cost-benefits of the scheme for the future, particularly with regard to the cost of the technology infrastructure (maintenance and replacement) against clinical health economic gains and prolonged use behavior of the system over time.

The eObs deployment is also associated with an approximate 50% reduction in reported EWS policy-related patient safety incidents in eObs wards. No such reduction was seen in non-eObs wards or in incident reporting in general over the same time period. Audit results also indicate adherence with EWS policy has improved because of the functionality of the system, namely, automated calculations, observation frequency setting, and user prompts that supports the findings from previous enquiries into electronic observations [17].

## Discussion

### Principal Findings

This evaluation has provided insight into the impact of mobile eObs and eHandover on working practice and elicited experiential data from the staff regarding their use of the new systems. From these data, a range of benefits to the hospital trust and workforce has been identified. The interview and focus group data in particular have also indicated where additional research and development could further benefit staff and patient experience [15].

The mobile solution has reduced EWS-related patient safety incidents and has allowed nurses and doctors to spend more time with the patient at the bedside. Internal studies of the paper and eObs processes for taking and recording a full set of observations showed a time saving of 1 min 23 seconds per patient using eObs. On the basis of 7500 sets of observations taken at NUH each day, this equates to approximately 170 hours of nursing time saved every day, releasing time to care. This aligns with Stevenson’s findings of how patient observations benefit from real-time capture at the point of care [18], and a reduction in nursing workload found by Wong et al [19].

Through personal ownership of devices, remote access has achieved real-time visibility of patient data across the whole hospital trust, allowing faster decision making and effective task prioritization. Clinicians are given vital information instantly because of automated escalations, and the need for multiple telephone calls is negated. This utility is further enhanced by users being able to access other medical apps and guidelines at the bedside. The eObs system appears to meet the strategies for EWS success identified by Russ *et al* [20] by being ubiquitous, being fit with ward workflow, and enabling records to be kept current and accessible.

**Figure 5:**
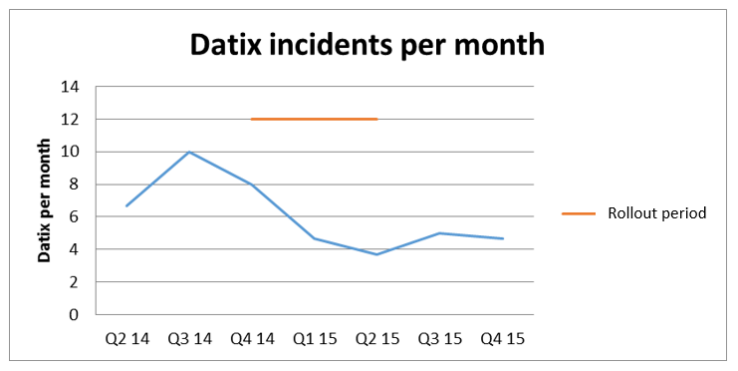
Early Warning Score–related incidents per month by quarter.

This transformation of practice has made it easier for staff to “do the right thing” even when not in attendance at the bedside. The opportunities provided through remote, distributed working practices have achieved safer working (see Figures 3-5) while not compromising communication, as evidenced in the interviews. The time previously spent searching for paper and chasing colleagues—delays similar to those reported by Fox and Elliot [21] in their examination of a paper-based EWS system—has been replaced with more meaningful discussions based on the information now visible through the mobile interface.

Previous work has demonstrated other wider benefits such as indicating to hospital managers which wards are particularly busy [22]. Where eObs has provided improved transparency about team workload and ward capacity, staff and system can begin working together as a joint cognitive system, which in turn has supported the implementation of smart resource allocation in times of pressure.

The study demonstrates how different clinical roles interact in the uptake and success of changes in practice or technology interventions. The role of junior staff as informal mentors and early adopters of new practices and technologies was evident, while senior staff backing was seen as crucial to success but was perceived to be lacking in this instance.

Challenges were also identified in technology integration and infrastructure, absence of feedback mechanisms for staff, management of expectations, and training requirements. Within the scope of the technology deployment, infrastructure issues were continually being encountered, evaluated, and improved upon, for example, in regards to Wi-Fi “blackspots” which disrupted eObs operations. However, the staff felt that there was a lack of investment in ICT support during the critical roll-out period, specifically during out-of-hours shifts. The eObs training occurred very rapidly, and the staff felt that more time to understand the system functionality would have been valuable. There was undisputed praise for the facilitation of the CICT team in conducting the technology roll-out, providing vital technology assistance and staff ICT interface on the ward during deployment—a strategy that the hospital has learnt from and will likely implement again. The issues reported in the evaluation about the absence of a feedback loop were considered and, in response, the eObs operational team developed a more transparent and accessible way for staff to provide feedback to them and ICT support. These findings provide commentary for organizational learning regarding future technology deployments.

This study provided evidence to show that appropriately designed and deployed HIT can support improved situation awareness with regard to patient deterioration. By combining eObs (a frequently accessed utility) with an eHandover system, staff have become used to entering data on mobile devices and are contributing to team-held data on clinical, patient safety, and operational issues. This model of HIT use and deployment is one which could assist future technology deployments in other hospitals and in doing so support the work of Cresswell et al [23] and Greenhalgh et al [24] in improving HIT implementation.

This research establishes 8 principles of good practice which can contribute to successful HIT deployments and which have been realized through this study.

1. Mobile tools to support clinical observation have the potential to be beneficial for doctors and nurses.
2. Deployment of this technology takes time, must involve working with users, and must be supported by a specialist technology deployment team.
3. More junior staff adapt to the technology particularly well.
4. Clinicians find ways of using this technology in conjunction with other tools to manage their work.
5. Embedded algorithms must take account of different specializations.
6. The technology can support clinical and patient communications.
7. It is vital that there is integration of new IT systems with existing systems.
8. The technology is only as good as the infrastructure that supports it.

### Limitations

The study data were limited to a single UK NHS Hospital Trust; however, the trust in question covers 2 sites and is the United Kingdom’s fourth largest acute trust.

This study and the deployment of the eObs system coincided with a rolling deteriorating patient improvement program at the host hospital trust. The direct observation period was also limited to 2 months postdeployment, and the data do not reveal if the staff settled back into old routines. As such, this should be the topic of a further enquiry to establish cause and effect in regards to the technology in isolation of other quality improvement initiatives.

There is the potential for response bias within the interview data. The views of those willing to participate (n=40) may not be representative of the wider workforce (approximately n=14,500). To combat this natural effect of the qualitative approach, the interviewer involved was an independent researcher not affiliated with the hospital, and the staff were sampled from a range of job roles, with varying levels of experience of patient bedside observations and data during a range of shifts.

### Future Work

There is opportunity to study the impact of further appropriation and expansion of eObs and specifically eHandover modules in clinical practice; analyze and measure the impact of improved situation awareness which is afforded by the technology and how to harness that information for effective workforce deployment and operational planning; investigate how mobile devices are being used on a personal level and where different clinical jobs and roles find utility in the technology so that this may be capitalized on and support further innovation [15].

Recent studies highlight the opportunities around continuous physiological monitoring of patients [25,26], utilizing technologies that are commonly used in HDUs. These solutions show positive results with regard to responding to patient deterioration but are costly and require detailed cost-benefit analysis to understand the health economic benefit of monitoring on such a large scale in hospitals. In relation to this type of personalized monitoring [27], eObs provides a potential step change, whereby the data gathered within the system could be exploited to understand trends within population and medical groups.

While this study focused on just 1 hospital in the United Kingdom, there is significant growth in this area, with hospitals in the United Kingdom [28] procuring through 2 main service providers, System C (previously Vitalpac)—supplying 26 NHS Trusts [29]—and Nervecentre 35 NHS Trusts, and also supplying health care providers in Sweden and Australia [30]. Electronic health records (EHRs) have a greater degree of maturity, with statistics from the United States suggesting that nearly as many as 80.5% of hospitals are using EHRs to some degree in their care provision [31]. Hospitals in other less-developed jurisdictions such as China [32] and India are also investing in those systems, with lessons learnt from India echoing some of the experiences examined in this study [33]. As such, there is much to gain from health care providers and manufacturers of these technology platforms in sharing the lessons learnt from such large-scale deployments to ease the transition from paper-based to electronic working and improve key outcomes with regard to quality measurement, staff performance, and patient experience and safety.

### Conclusions

The eObs and eHandover project has effected transformational changes in patient safety at a large acute hospital, bringing benefit to both staff and patients. In the hands of clinical staff, handheld devices and appropriate clinical software have the potential to reduce costs associated with inpatient management while simultaneously empowering staff in their daily activities, improving patient safety and releasing time to care.

Looking ahead, the full scale of the benefits experienced by this hospital trust is only just beginning to mature, with their full extent being realized.

## Abbreviations

CCOT: critical care outreach team
CICT team: Clinical Information and Communication Technology team
eObs: electronic observations
eHandover: electronic handover
EWS: early warning score
HDU: high dependency unit
HIT: health information technology
ICU: intensive care unit
ICT: intermediate care team
IT: information technology
NHS: National Health Service
NUH: Nottingham University Hospitals
PC: personal computer
